# Nano-enabled strategies for targeted immunotherapy in gastrointestinal cancers

**DOI:** 10.3389/fimmu.2025.1653829

**Published:** 2025-08-14

**Authors:** Chaofan Chen, Jinlei Li, Xiaokun Hua, Tingting Deng, Zhiyun Zhang

**Affiliations:** ^1^ Department of Anorectal, Kunming Municipal Hospital of Traditional Chinese Medicine, The Third Affiliated Hospital of Yunnan University of Chinese Medicine, Kunming, Yunnan, China; ^2^ Department of Orthopedics, Kunming Municipal Hospital of Traditional Chinese Medicine, The Third Affiliated Hospital of Yunnan University of Chinese Medicine, Kunming, Yunnan, China

**Keywords:** gastrointestinal cancers, nanomedicine, tumour microenvironment, precision oncology, immunotherapy

## Abstract

Gastrointestinal (GI) cancers remain a leading cause of global cancer morbidity and mortality, demanding novel therapeutic strategies that overcome existing limitations. Nanomedicine has recently emerged as a transformative approach, offering the potential to significantly enhance immunotherapy outcomes through precision targeting and modulation of tumour immune microenvironments. This review discusses the principal categories of precision-engineered nanoparticles—including lipid-based carriers, polymeric systems, protein-derived formulations, and metallic-hybrid composites—emphasising their capacity for targeted immune modulation and improved pharmacokinetic profiles. These nanoparticle platforms strategically intervene across multiple stages of the cancer-immunity cycle, facilitating antigen presentation, T-cell activation, and cytotoxic lymphocyte infiltration, and augmenting immune checkpoint blockade efficacy. Clinically approved nanoformulations such as Abraxane, Doxil, Onivyde, and emerging mRNA-based nanovaccines highlight promising translational outcomes in GI malignancies, demonstrating improved therapeutic indices and reduced systemic toxicity. Nonetheless, clinical implementation remains challenged by nanoparticle complexity, heterogeneous tumour biology, clearance mechanisms, and toxicity concerns. Future success will depend on integrated strategies combining advanced nanoparticle engineering, precise administration routes, rigorous translational validation, and rational therapeutic combinations to realise the full potential of nanomedicine-based immunotherapies in gastrointestinal oncology.

## Introduction

Worldwide, gastrointestinal (GI) tract cancers accounted for 26.0% of all new cancer diagnoses and 35.0% of cancer-related deaths in 2018 (1). By 2040, the global incidence of GI cancers is projected to rise to approximately 7.5 million cases annually, resulting in an estimated 5.6 million deaths (2). In 2020, the lifetime probability of developing and dying from a GI malignancy was 8.20% (95% CI 8.18–8.21) and 6.17% (95% CI 6.16–6.18), respectively. Colorectal cancer constitutes the largest share of this burden, accounting for 38.5% of lifelong incidence and 28.2% of GI cancer mortality, followed by cancers of the stomach, liver, oesophagus, pancreas, and gallbladder (3).

Despite therapeutic advances, cancer continues to impose a substantial global health burden, compelling ongoing efforts to develop novel preventive, therapeutic, and supportive strategies to enhance patient outcomes (4). Immunotherapy has transformed the oncology landscape by leveraging endogenous immune mechanisms to generate potent and targeted antitumour responses, often demonstrating reduced toxicity compared to conventional chemotherapy (5, 6). These biologic or synthetic immunotherapeutic agents stimulate innate and adaptive immune pathways to counteract immune-evasive mechanisms exploited by tumours. As such, immunotherapies hold promise for achieving durable remission and, in some cases, complete tumour eradication. However, precise immune regulation remains challenging, as these therapies may induce significant immune-related adverse events, including autoimmunity and systemic inflammation (7).

Nonetheless, clinical outcomes from immunotherapy remain constrained by suboptimal response rates, limited therapeutic efficacy, and safety concerns (8, 9). Many immunotherapeutics suffer from unfavourable pharmacokinetic profiles, characterised by poor solubility, inadequate stability, and rapid clearance, thus limiting sustained therapeutic effectiveness (10). Moreover, severe adverse reactions, such as hypersensitivity and inflammatory responses, occasionally result in serious or fatal outcomes (11). The immunosuppressive tumour microenvironment (iTME) further restricts the effective delivery and activation of immune agents, while the inherent low immunogenicity of tumours, alongside the accumulation of regulatory T cells, myeloid-derived suppressor cells, and suppressive cytokines within the iTME, collectively undermine immunotherapy’s efficacy (12, 13).

GI malignancies exhibit distinct biological characteristics that influence their immunotherapeutic responses. Colorectal, gastric, pancreatic, and oesophageal cancers generally present immunologically “cold” tumour microenvironments (TME), characterised by low immune cell infiltration, enriched immunosuppressive populations such as regulatory T cells (Tregs), myeloid-derived suppressor cells (MDSCs), and tumour-associated macrophages, along with dense fibrotic stroma. These collectively impede effective immune cell infiltration and activation, significantly limiting anti-tumour immunity (14). Current immunotherapy, largely based on programmed cell death-1 (PD-1)/PD-ligand 1 (PD-L1) blockade, has achieved notable but moderate successes in GI cancers. Phase 3 clinical trials have validated PD-1 inhibitors such as nivolumab for advanced gastric cancer and pembrolizumab, nivolumab, tislelizumab, and camrelizumab for advanced oesophageal cancer, yet objective response rates (ORR) hover around only 15% (15). Particularly in metastatic colorectal cancer (mCRC), the therapeutic benefit of anti-PD-1 therapy is restricted predominantly to microsatellite instability-high (MSI-H) or mismatch repair-deficient (dMMR) subsets, whereas patients with microsatellite stable (MSS) or proficient mismatch repair (pMMR) status, comprising approximately 95% of the mCRC population, exhibit response rates as low as 0–2% (16). Such modest clinical outcomes highlight the necessity for novel strategies to overcome intrinsic biological and therapeutic barriers in GI malignancies.

Nano-enabled immunotherapy offers a complementary solution by improving pharmacokinetics and biodistribution, co-packaging synergistic immunomodulators, and delivering them with spatial and temporal precision to remodel the GI tumour microenvironment. Rationally designed nanoparticles can enhance antigen preservation and cross-presentation, boost T-cell priming and intratumoural trafficking, induce or amplify immunogenic cell death, and activate innate immune pathways (e.g., STING/TLR) while limiting systemic toxicity (17–19). In parallel, theranostic nanoparticle platforms integrate radiosensitisation and real-time molecular/immunologic imaging, enabling response-adapted combinations and dosing in GI cancers (20–22). In particular, Li et al. have comprehensively reviewed how nanoparticles can potentiate radio-immunotherapy by amplifying radiation-induced immunogenic cell death, enhancing dendritic-cell cross-presentation of tumour antigens, and remodeling the tumour immune microenvironment—thereby fostering robust abscopal effects and durable antitumour immunity (23). Complementarily, biomarker-driven molecular imaging probes—such as PET tracers for PD-L1 expression and CD8^+^ T-cell infiltration—have been developed to noninvasively monitor immune activation and radiation response *in vivo*, supporting patient stratification and real-time optimisation of combined radio-immunotherapy regimens (21).

Nanotechnology-based immunotherapies have emerged as a promising approach, strategically intervening at distinct stages of the cancer–immunity cycle through four conceptual modules (1): tumour antigen release and presentation, where engineered nanoparticles such as mesoporous silica nanoparticles protect endogenous tumour antigens from clearance and enhance lymphoid tissue delivery, as exemplified by the work of Qian et al. (24); (2) T-cell priming and activation, with artificial antigen-presenting cells (aAPCs) optimised at the nanoscale for efficient lymph-node targeting and costimulatory activation—though particle size critically influences efficacy, as demonstrated by Hickey et al. (25); (3) cytotoxic T-lymphocyte (CTL) trafficking and infiltration, where nanocarriers encoding gene therapies like chemokine “traps” modulate chemotactic signals to enhance T-cell infiltration, as illustrated by Goodwin et al. (26) Moreover, in orthotopic colorectal models, STING-activating nanosystems reduce tumour hypoxia and upregulate endothelial ICAM-1/VCAM-1 expression, resulting in a twofold increase in CD8^+^ T-cell tumour infiltration and improved survival (27); and (4) CTL recognition and tumour cell killing, involving nanoparticles engineered for enhanced checkpoint inhibitor avidity and retention, such as generation-7 PAMAM dendrimer conjugates reported by Bu et al. (28) Additionally, combinatorial nano-immunotherapies integrate complementary agents acting across multiple immunologic stages, addressing the limitations of monotherapy and enabling synergistic antitumour effects.

## Representative GI cancer models and platforms

To evaluate nano-enabled immunotherapies in relevant settings, we summarise here the most widely used *in vivo* systems for each major GI cancer subtype (**Table 1**):

**Table 1:** Representative preclinical models for gastrointestinal cancers.

GI cancer type	Model	Features	Reference
Colorectal Cancer –CT26 (BALB/c) syngeneic model	CT26 (BALB/c)	Chemically induced carcinoma; “hot” phenotype with robust CD8^+^ T-cell infiltration; sensitive to PD-1 blockade.	(29)
	MC38 (C57BL/6) syngeneic model	MSI-high model; recapitulates hypermutated CMS1 tumours; responsive to PD-1/PD-L1 inhibition.	(38)
Gastric Cancer	MKN-45 orthotopic xenograft	Human gastric adenocarcinoma line injected into stomach wall of NSG mice; reproduces primary growth and metastatic spread to liver/peritoneum.	(32)
Pancreatic Cancer	KPC GEMM	LSL-Kras^G12D; LSL-Trp53^R172H; Pdx1-Cre mice develop spontaneous PDAC with dense desmoplastic stroma and an immunosuppressive TME.	(39)
	Orthotopic KPC cell transplant	KPC-derived cells implanted into pancreas of C57BL/6 mice; preserves stromal interactions; suitable for rapid therapeutic assessment.	(40)
Humanised Models	PBMC-engrafted NSG mice	NSG mice reconstituted with human PBMCs; evaluate human-specific immunotherapies (e.g., CAR-T, bispecific antibodies) *in vivo*.	(41)

Colorectal Cancer (CRC): CT26 (BALB/c) and MC38 (C57BL/6) syngeneic models, modeling MSI-high and “hot” CRC phenotypes, respectively, and routinely used to assess PD-1/PD-L1 blockade efficacy (29, 30).

Gastric cancer (GC): Orthotopic engraftment of human MKN-45 or NUGC-4 cells into NSG mice reproduces primary tumour growth, metastatic spread, and immune exclusion, providing clinically relevant platforms (31, 32).

Pancreatic ductal adenocarcinoma (PDAC): The KPC GEMM (LSL-Kras^G12D; LSL-Trp53^R172H; Pdx1-Cre) develops spontaneous, desmoplastic PDAC with immunosuppressive stroma. Orthotopic transplantation of KPC-derived tumour cells into syngeneic hosts offers rapid evaluation of therapeutic interventions (33, 34).

Humanised platforms: PBMC-engrafted NSG and HLA-transgenic NSG mice permit *in vivo* testing of human-specific immunotherapies (e.g., CAR-T cells, bispecific antibodies), enabling translational assessment of nano-enabled strategies (35, 36). For example, PLGA nanoparticles encapsulating STING agonists (e.g., ONP-302) in PBMC-engrafted NSG mice elicit IL-15–dependent activation of human NK cells and CD8^+^ T cells, demonstrating significant tumour growth suppression and translational relevance (37).

## Nanomaterial-facilitated immunostimulation in gastrointestinal malignancies

**Figure 1:**
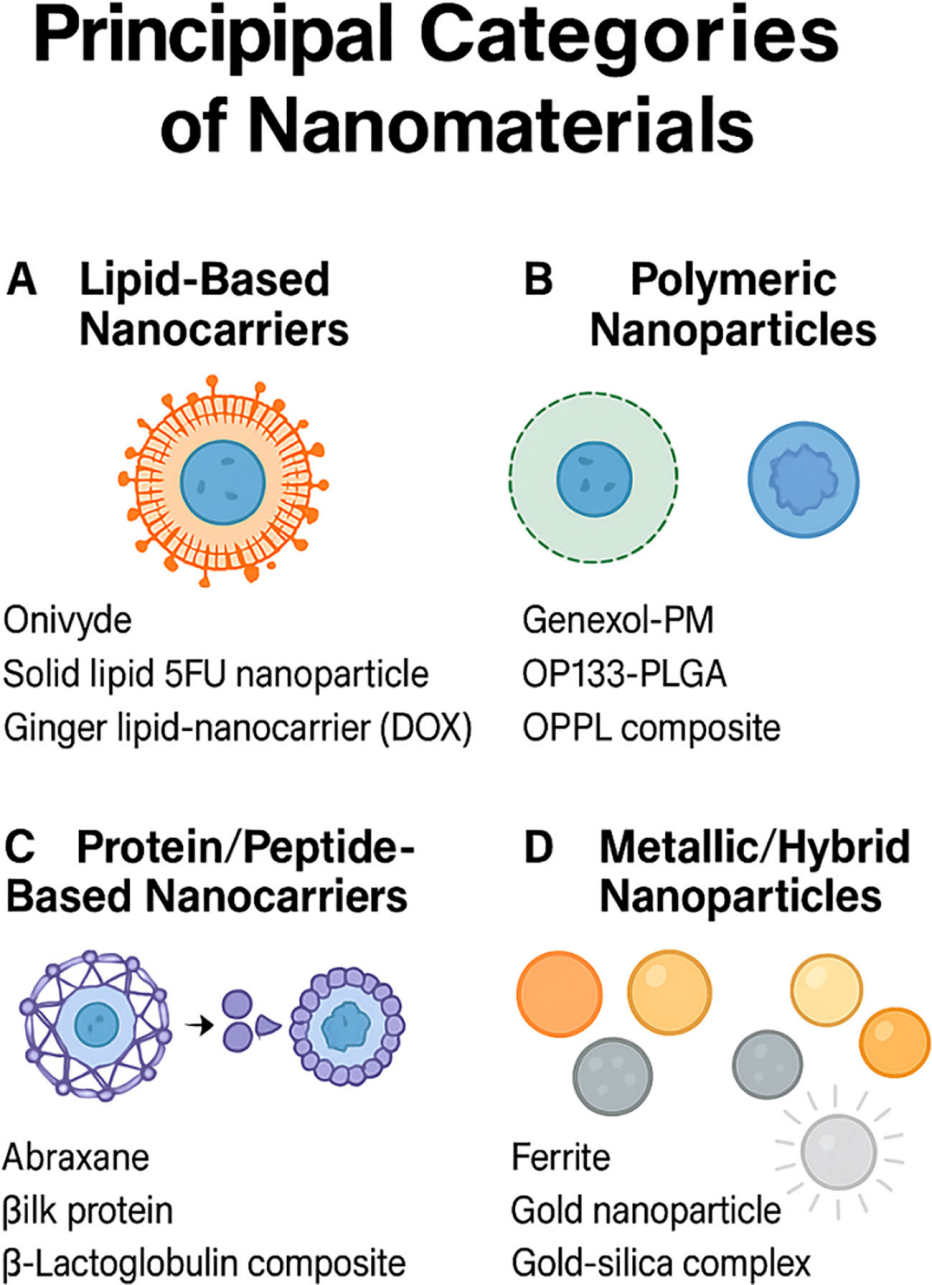
Principal categories of nanomaterials for drug delivery. **(A)** Lipid‐based nanocarriers such as liposomes and solid‐lipid particles (Onivyde; solid‐lipid 5-FU nanoparticle; ginger lipid carrier loaded with doxorubicin). **(B)** Polymeric nanoparticles including Genexol-PM, OP133-PLGA, and OPPL composites. **(C)** Protein/peptide assemblies exemplified by Abraxane, silk protein, and β-lactoglobulin composites. **(D)** Metallic and hybrid nanoparticles such as ferrite, gold, and gold–silica complexes.

Originally designed to enhance pharmacokinetic profiles and improve tumour specificity, several clinically approved nanomedicines have found renewed relevance as immunomodulatory tools in GI oncology (42, 43). Recent studies have demonstrated that various nanomaterial platforms can significantly enhance immunostimulatory effects within GI tumour models. For instance, mesoporous silica nanoparticles and metal-based nanoparticles have shown potent induction of immunogenic cell death (ICD), characterised by calreticulin (CRT) exposure, extracellular ATP release, and HMGB1 secretion, collectively enhancing dendritic cell maturation and subsequent T-cell priming (44). Herein, we outline principal categories of nanomaterials (**Figure 1**), briefly noting their approved indications within GI cancers, before examining how these platforms have been re-engineered to engage both innate and adaptive immune responses within the tumour microenvironment.

### Lipid-based nanocarriers

Lipid-based systems, particularly liposomes and lipid nanoparticles (LNPs), represent the most clinically pervasive nanotechnologies in oncology, attributable to their favourable safety profiles, modular drug-loading capacities, and scalability in production (45). Among GI indications, the PEGylated liposomal formulation of irinotecan (Onivyde), licensed for metastatic pancreatic cancer, exemplifies this class (46). By promoting tumour-specific accumulation and extended plasma half-life, it not only augments cytotoxicity but also triggers immunogenic cell death, facilitating the liberation of neoantigens for dendritic cell engagement and cross-priming.

The clinical utility of LNPs was cemented by the 2018 FDA approval of ONPATTRO, the first liver-targeting nanoparticle for RNA delivery (47). More compact liposomal LNP–mRNA formulations rich in bilayer lipids—egg sphingomyelin and cholesterol—achieve >90% encapsulation, extended circulation, and efficient extrahepatic transfection via pH-triggered core release (48).

Investigational liposomal oxaliplatin formulations under evaluation in colorectal cancer similarly exploit these immunogenic properties, inducing calreticulin exposure and ATP release (49, 50). Juang and colleagues further engineered pH-sensitive, peptide-functionalised LNPs co-loaded with irinotecan and miR-200, a microRNA known to inhibit metastatic progression (51). Given its widespread use in colorectal cancer, 5-fluorouracil (5-FU) remains a key chemotherapeutic agent, despite its short half-life and systemic toxicity. To address these pharmacological limitations, Patel et al encapsulated 5-FU within solid lipid nanoparticles, demonstrating enhanced cytotoxicity in Caco-2 cells in a dose-dependent manner (52).

Doxorubicin (DOX), though potent, is hindered by its cardiotoxicity and poor tumour selectivity. To overcome these drawbacks, Zhang et al utilised nanovectors derived from ginger lipids (GDNVs) to encapsulate and deliver DOX efficiently to tumour sites, improving therapeutic precision and limiting off-target effects (53).

### Polymeric nanoparticles

Polymeric nanostructures, including nanospheres and nanocapsules, offer a highly customisable platform for drug delivery, enabling responsive release profiles, environmental stability, and surface functionalisation for targeted therapy (54, 55). These systems can exploit pathophysiological features of the tumour milieu—such as acidic pH and elevated enzymatic activity—to trigger site-specific drug liberation (56–58).

Genexol-PM, a paclitaxel-loaded polymeric micelle approved for breast and pancreatic malignancies, exemplifies how prolonged drug exposure may elicit immunogenic cell death in GI models (59). In colon cancer models, chitosan-coated PLGA nanoparticles encapsulating 5-FU markedly suppressed HT-29 cell viability compared to free drug or uncoated formulations (60). Further innovation has yielded a multifunctional nanocomposite—OPPL—integrating oleic acid-modified superparamagnetic iron oxide with an amphiphilic polymer for the co-delivery of a platinum prodrug and lauric acid, aiming to synergistically amplify colorectal tumour cytotoxicity and disrupt microbial biofilms (61).

In recent preclinical work, polyguanidine-derived nanoinhibitors have been engineered to localise within hepatocyte lysosomes and inhibit V-ATPase activity, thereby activating AMPK signalling to reduce fatty acid synthesis and enhance lipolysis in models of liver lipid overload (62). Equally promising are enzyme-responsive branched glycopolymer nanoassemblies that, upon cathepsin B–mediated cleavage in gastric cancer cells, co-release paclitaxel and an Akt inhibitor to achieve potent synergistic tumour kill with mitigated systemic toxicity (63).

### Protein- and peptide-based nanocarriers

Nanoparticles derived from natural proteins—such as albumin, silk fibroin, gelatin, and lipoproteins—are increasingly explored for cancer nanomedicine owing to their intrinsic biodegradability, low immunogenicity, and high drug-loading capacity (18, 64).

Albumin-bound paclitaxel (Abraxane), while approved for various solid tumours, has demonstrated promise in GI cancers when co-administered with immune checkpoint inhibitors, fostering dendritic cell maturation and macrophage repolarisation via receptor-mediated endocytosis (65). Recent advances include the development of folate-conjugated sericin nanoparticles (FA-SND), engineered for pH-responsive doxorubicin release and selective accumulation in tumour tissues (66). Another innovative construct, based on a GE11-HGFI fusion protein, enabled the targeted delivery of curcumin to EGFR-overexpressing colorectal cancer cells, yielding enhanced intratumoural drug retention and pronounced anticancer activity (67). β-Lactoglobulin nanoparticles co-loaded with 5-FU and sodium butyrate, and functionalised with folic acid, were found to selectively home to folate receptor–expressing colorectal tumours, offering a strategy for combined chemotherapeutic and epigenetic modulation (68).

### Metallic and hybrid nanoparticles

While iron oxide–based agents such as NanoTherm are approved for thermotherapy in glioma and prostate cancer (69), their application has been adapted for GI tumours, particularly colorectal cancer. In preclinical settings, iron oxide nanoparticles co-administered with TLR agonists generated reactive oxygen species and heightened dendritic cell activation, contributing to local immune potentiation (70). Recent studies have extended these approaches: artemisinin−protected magnetic iron−oxide nanoparticles hyperactivate autophagy to overcome hyperthermia resistance in gastric cancer, while glucose−coated Fe₃O₄ nanoparticles conjugated with safranal selectively induce apoptosis and G₂/M cell−cycle arrest in hepatocellular carcinoma models (71, 72).

Hybrid nanoparticles—featuring gold cores for photothermal ablation and polymeric coatings harbouring STING agonists—have yielded synergistic effects in orthotopic pancreatic cancer models, enhancing both innate immune engagement and antigen release (73). In MC38-bearing mice, dual-STING agonist nanoparticles (STANs) have been shown to elevate intratumoural IFN-β by more than threefold and increase CD86^+^ dendritic cell frequencies, correlating with enhanced CD8^+^ T-cell infiltration and durable antitumour responses (74). Variations in gold nanoparticle systems, including PEGylated AuS, have exhibited substantial photothermal efficiency and anti-tumour effects in gastric cancer (75, 76). Nanosystems such as FAL-ICG-HAuNS not only exert photothermal cytotoxicity but also elicit robust immune responses *in vivo* (77). Moreover, composite constructs integrating gold nanorods with multi-walled carbon nanotubes enable enhanced imaging in gastric tumours (78), while folate-targeted silica-encapsulated gold nanoclusters demonstrate both diagnostic and therapeutic utility, offering precise tumour localisation and treatment monitoring (79). Beyond these, multifunctional metallic−hybrid platforms—such as pH−responsive Fe−doped mesoporous polydopamine co−loaded with sorafenib for synergistic ferroptosis and photothermal therapy, ultrathin 2D FeS nanosheets delivering CRISPR/Cas9 to downregulate heat−shock proteins, and self−assembled copper−based nanoplatforms enabling chemodynamic, photodynamic, and antiangiogenic tritherapy—have shown potent multimodal cytotoxicity across diverse GI malignancy models (80–82).

## Nanomaterial-enhanced immunotherapeutic strategies in gastrointestinal cancers: mechanisms and clinical translation

**Figure 2:**
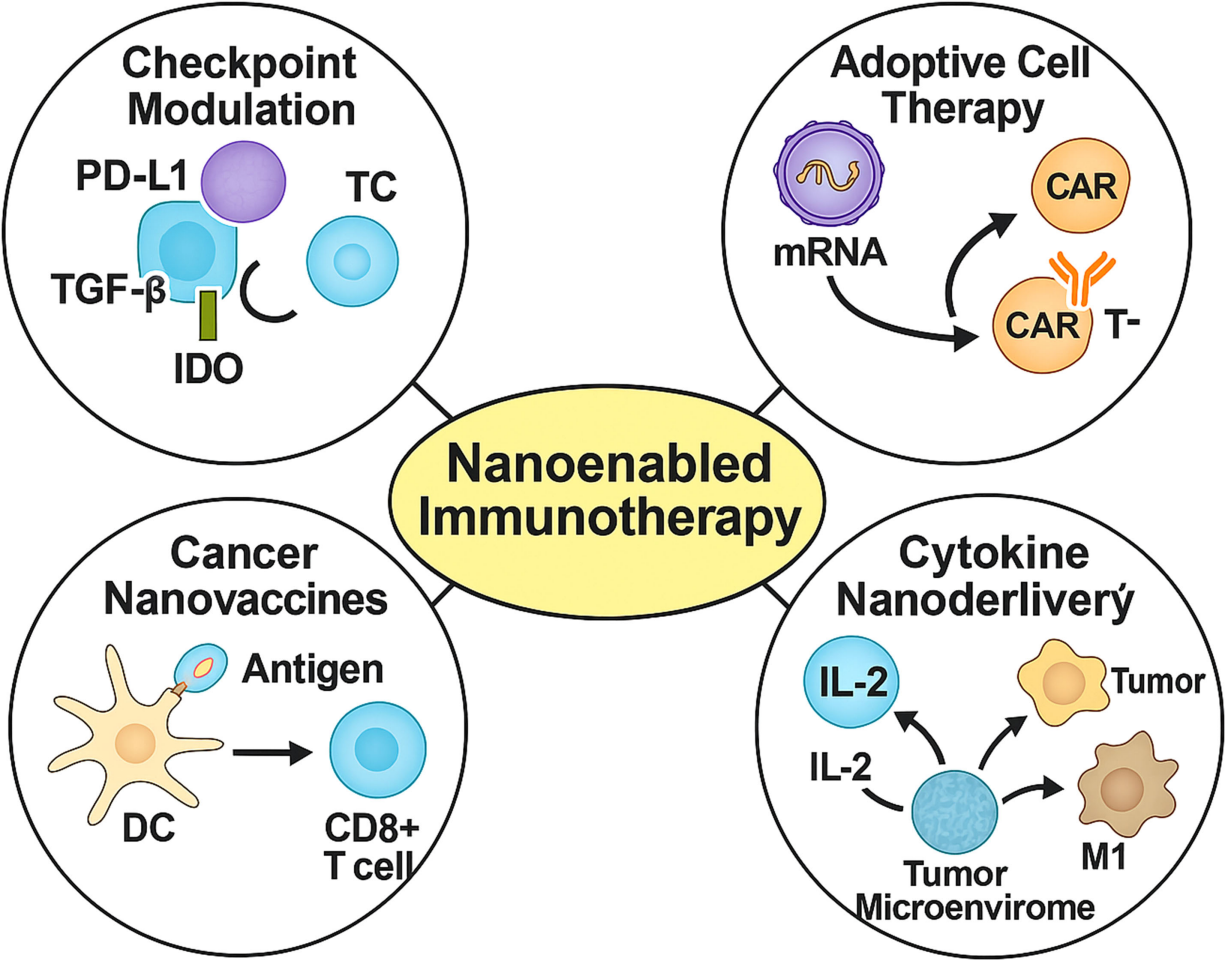
Four representative nanomedicine-enabled immunotherapeutic strategies for gastrointestinal cancers. Nanoparticle platforms facilitate checkpoint inhibition (e.g., PD-L1, TGF-β, IDO), *in vivo* programming of CAR-T or NK cells, dendritic cell-mediated vaccination, and targeted cytokine delivery (e.g., IL-2, IL-12), enhancing tumour immune infiltration, cytotoxicity, and therapeutic selectivity.

Nanotechnology offers transformative potential for GI cancer immunotherapy by integrating enhanced permeability and retention effects with active targeting, deep tissue penetration, and controlled release kinetics. Functionalisation with antibodies or ligands allows for precise delivery of immunotherapeutics—ranging from checkpoint inhibitors to cytokines and neoantigens—enabling reprogramming of the tumour immune microenvironment while limiting systemic toxicity (18). Here, we highlight four key nanotechnology-enabled strategies (**Figure 2**): precision checkpoint blockade, adoptive cell therapy enhancement, nanovaccines, and cytokine delivery.

### Precision immune checkpoint modulation

**Figure 3:**
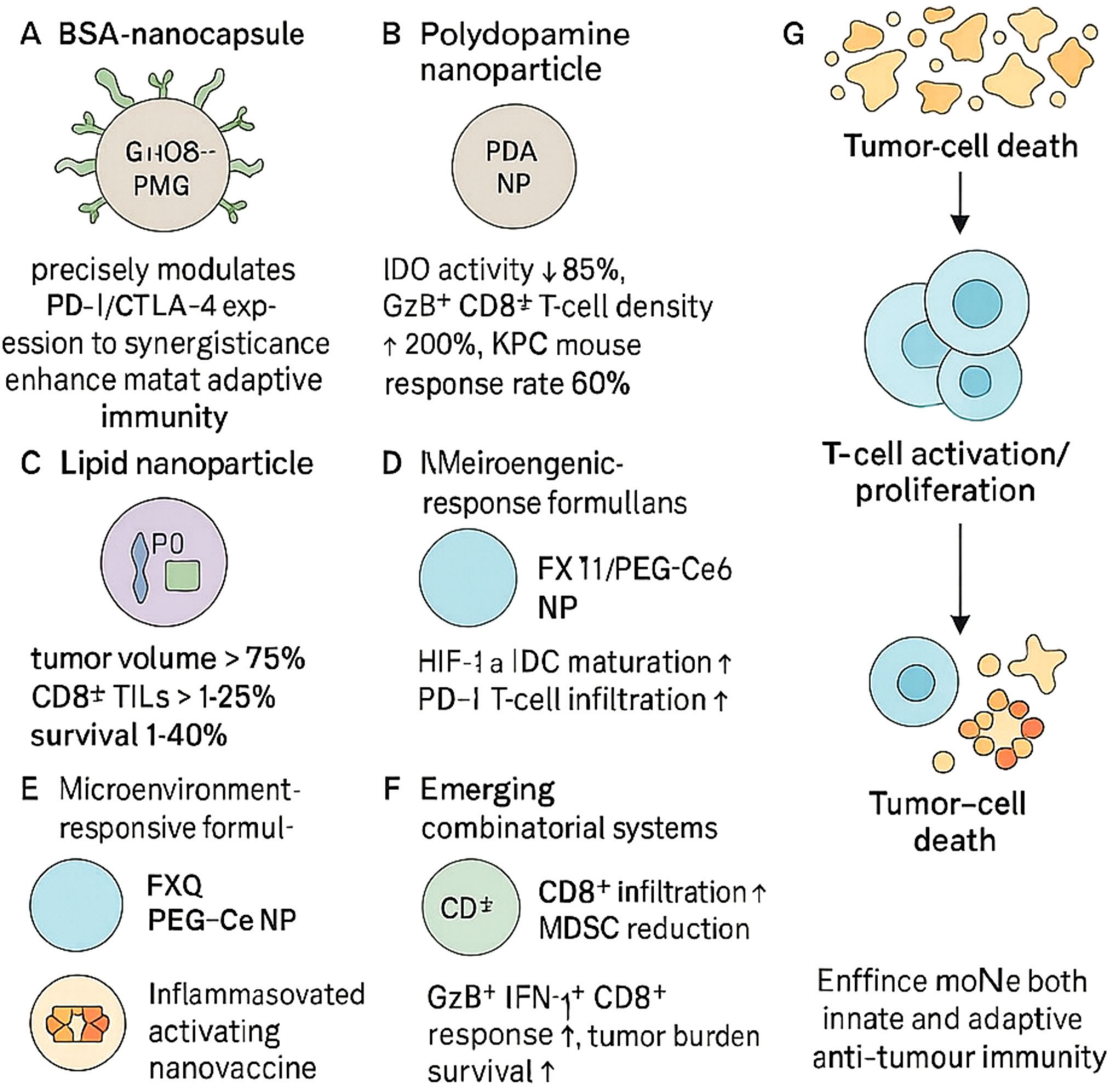
Nanoparticle-based precision immune checkpoint modulation enhances antitumour immunity. Engineered nanoparticles modulate immune checkpoints to overcome tumour-induced immunosuppression and promote effective immune responses. **(A)** BSA nanocapsules enhance PD-1/CTLA-4 regulation and adaptive immunity. **(B)** Polydopamine nanoparticles reduce IDO activity and increase CD8^+^ T-cell infiltration, improving response rates in pancreatic models. **(C)** Lipid nanoparticles restore CD8^+^ TILs and reduce tumour burden. **(D, E)** Microenvironment-responsive formulations alleviate hypoxia or acidity to enhance dendritic cell and T-cell function. **(F)** Combinatorial systems further boost CD8^+^ T-cell responses and reduce MDSCs. **(G)** Together, these strategies promote tumour cell death through enhanced innate and adaptive immunity.

Nanoparticles can be tailored to modulate immune checkpoints on tumour or immune cells, thus promoting robust innate and adaptive immune responses (**Figure 3**). One such system involves BSA-based nanocapsules functionalised with glucose-modified PMPC polymers and IgG, achieving precise immune regulation (83). In another study, polydopamine nanoparticles co-loaded with gemcitabine and the IDO inhibitor NLG919 suppressed intratumoural IDO activity by 85%, doubled intratumoural granzyme B^+^ CD8^+^ T-cell density, and induced durable responses in 60% of KPC mice, outperforming gemcitabine alone (10%) (84).

Lipid-based nanoparticles co-encapsulating PD-L1 siRNA and a TGF-β receptor antagonist reduced tumour volumes by over 75% in pancreatic cancer models and restored CD8^+^ T-cell infiltration to >25% of tumour-infiltrating lymphocytes, prolonging survival by 40% compared with free drugs (85). Chemotherapy-induced tumour RNA nanoparticles synergised with PD-1 blockade to suppress colorectal tumour progression and prolong survival (86). Similarly, oxaliplatin-loaded liposomes combined with anti-PD-1 achieved 90% tumour growth inhibition in CT26 models—substantially surpassing monotherapies (30–50%) via enhanced immunogenic cell death and antigen cross-presentation (85).

Recent microenvironment-responsive formulations have further refined checkpoint modulation. In gastric cancer, FX-11@PEG-Ce6 nanoparticles alleviated TME acidity, promoted dendritic cell maturation and CD8^+^ T-cell cytokine secretion, and markedly enhanced the efficacy of α-PD-1 therapy *in vivo* (87). Hypoxia-activated TH-302 encapsulated in mPEG-PLGA NPs selectively released under low-oxygen conditions, reduced HIF-1α and PD-L1 expression, and, in combination with PD-1 blockade, significantly increased CD8^+^ T-cell infiltration and pro-inflammatory cytokine production in gastric tumour models (88). In hepatocellular carcinoma, pH-sensitive nanocarriers co-delivering anti-PD-1 and MDK-siRNA reprogrammed TAMs and MDSCs towards an immunostimulatory phenotype, overcoming ICB resistance (89). Lastly, AEAA-targeted PLGA nanoparticles co-encapsulating mitoxantrone and the STAT3 inhibitor napabucasin activated cGAS-STING signalling in HCC cells, synergised with anti-PD-1, and achieved durable tumour regression (90).

A nanoparticle system carrying siRNAs against ZDHHC9 enhanced CD8^+^ T-cell infiltration and reduced MDSC presence, converting the immunosuppressive tumour microenvironment into a pro-inflammatory one when combined with PD-L1 blockade (91). Inflammasome-activating nanovaccines also promoted GzB^+^IFN-γ^+^ CD8^+^ T-cell responses and synergised with CTLA-4/PD-L1 inhibitors to reduce tumour burden and extend survival across various murine cancer models (92).

### Nano-enabled adoptive cell therapies

**Figure 4:**
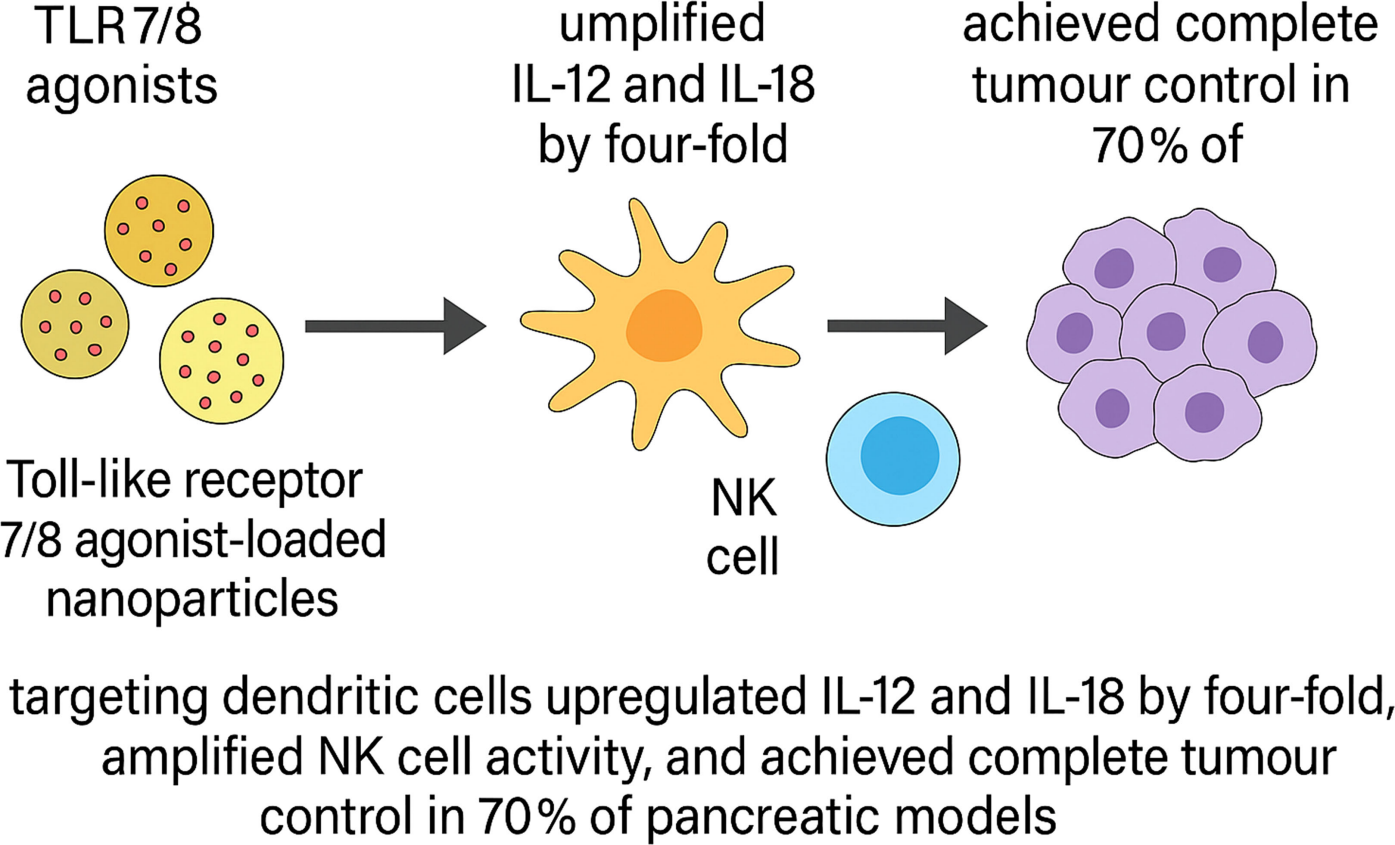
Schematic illustration of TLR7/8 agonist-loaded nanoparticle–mediated innate immune activation and tumour control in pancreatic cancer models. Toll-like receptor 7/8 (TLR7/8) agonist-loaded nanoparticles selectively target dendritic cells, leading to a four-fold upregulation of proinflammatory cytokines IL-12 and IL-18. These cytokines promote natural killer (NK) cell activation, resulting in enhanced cytotoxic activity. In preclinical pancreatic tumour models, this strategy achieved complete tumour regression in 70% of treated animals.

Nanomaterials have been harnessed to augment the expansion, homing, and persistence of engineered lymphocytes within fibrotic GI tumours. One approach used antibody-decorated ionisable lipid nanoparticles to deliver CD19-CAR mRNA systemically, inducing ~35% CAR expression in circulating T cells, with functional persistence and >90% target B-cell depletion in mice (93). Toll-like receptor 7/8 agonist-loaded nanoparticles targeting dendritic cells upregulated IL-12 and IL-18 by four-fold, amplified NK cell activity, and achieved complete tumour control in 70% of pancreatic models (**Figure 4**) (94).An injectable alginate-based nanogel releasing CCL21 chemokine enhanced CD8^+^ T-cell and dendritic cell infiltration five-fold and reduced metastatic spread in colorectal models (95).

Building on this foundation, a STAR-based *in vivo* selection yielded a CEA-specific nanobody (VHHB30); consequently, third-generation VHHB30–CAR T cells eradicated colorectal and gastric xenografts and outperformed second-generation constructs (96). In parallel, LNP platforms have emerged as versatile tools to transiently program immune cells with high efficiency and low cytotoxicity: in HCC models, a bespoke lipid blend directed CAR mRNA–loaded LNPs to liver-associated myeloid cells, thereby enhancing local delivery (97); moreover, Billingsley’s top-performing C14-4 ionizable LNP enabled robust CAR mRNA transfection of primary human T cells (98), and antibody-decorated LNPs (anti-CD3 Ab-LNPs) further improved T-cell targeting (99).

### Nanovaccines in GI oncology

**Figure 5:**
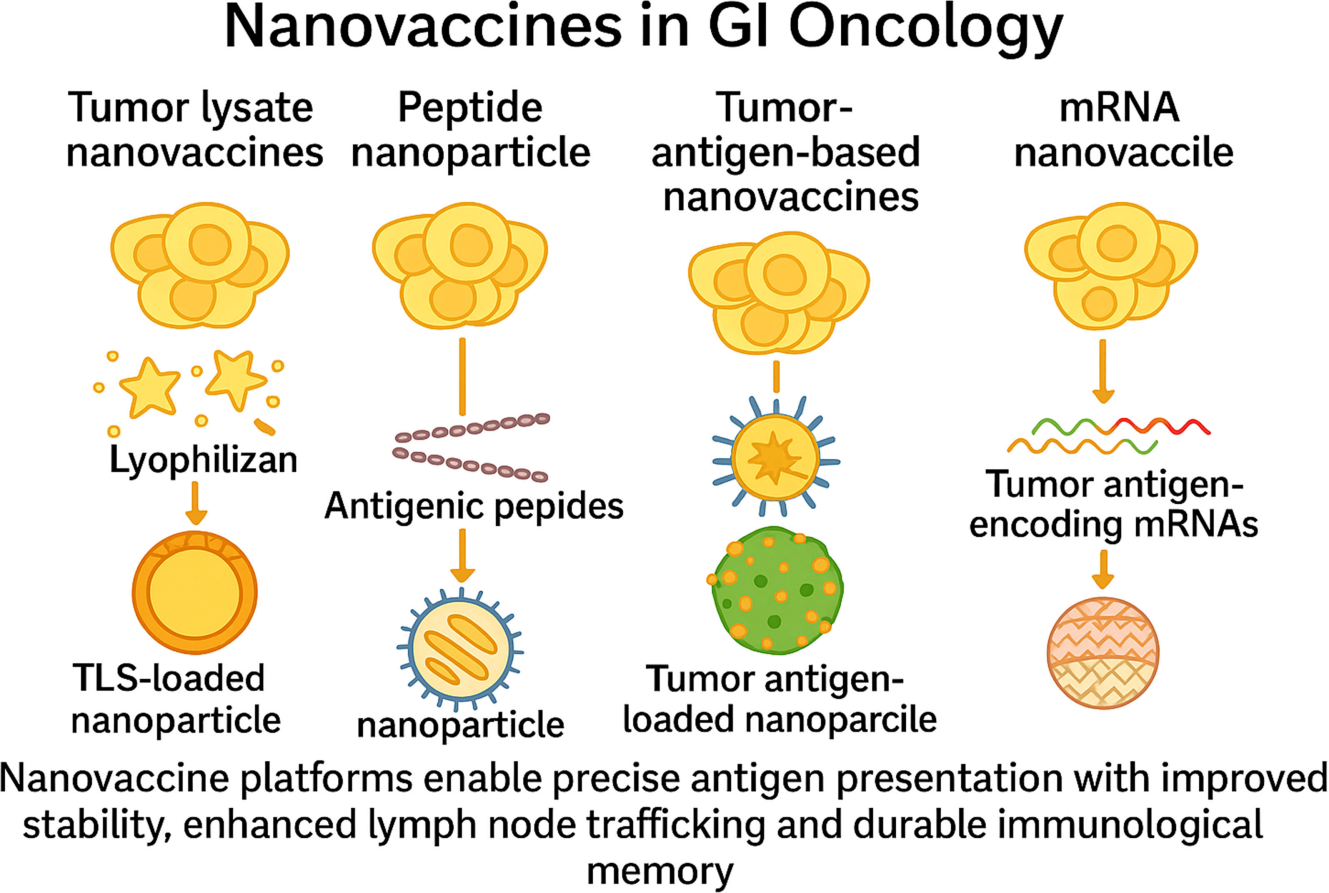
Nanovaccine platforms in gastrointestinal oncology. This figure depicts four nanovaccine strategies used in GI cancers. Tumour lysate nanovaccines deliver whole-cell antigens via nanoparticle encapsulation. Peptide nanovaccines incorporate defined antigenic peptides to elicit specific T-cell responses. Tumour antigen-based nanovaccines co-deliver tumour antigens and adjuvants to enhance dendritic cell activation. mRNA nanovaccines encode tumour antigens for *in situ* expression. All platforms aim to enhance antigen stability, lymph node trafficking, and durable immune memory.

Nanovaccine platforms enable precise antigen presentation with improved stability, enhanced lymph node trafficking, and durable immunological memory (**Figure 5**) (100–102). Antigen payloads may include peptides, tumour lysates, or mRNA, aiming to broaden the T-cell repertoire and overcome immunosuppression (103, 104).

In hepatocellular carcinoma, a phase I/II study of the DNA vaccine GNOS-PV02 plus pembrolizumab achieved an objective response rate >30%, exceeding historical anti-PD-1 monotherapy benchmarks (14–17%) and underscoring the need for larger confirmatory trials (105). A smart, tumour-microenvironment–responsive Fe/Mn nanovaccine that synchronously releases sorafenib, Fe³^+^ and Mn²^+^ triggered pyroptosis-mediated immunogenic cell death and cGAS–STING activation, driving dendritic-cell maturation and potent antitumour immunity (106). Complementarily, an *in situ* layered double hydroxide–cGAMP nanovaccine deployed after radiofrequency ablation adsorbed tumour-associated antigens, boosted type I interferon signalling, increased CTL/DC infiltration, and markedly enhanced αPD-L1 efficacy in poorly immunogenic liver tumours (107).

A lipopolyplex-based mRNA vaccine encoding CT26 neoantigens, co-delivered with CpG-ODN, induced IFN-γ responses in >80% of neoantigen-specific CD8^+^ T cells and achieved complete tumour regression in 80% of mice, with 90% protected against rechallenge (108). Similarly, biodegradable polymeric nanoparticles encoding KRAS^G12D mRNA eradicated MC38 and CT26 tumours while inducing long-term memory responses (109).

Engineered Salmonella coated with tumour lysate–adsorbed PDA nanoparticles (EnS@PDA@CL) homed to hypoxic tumour regions and activated dendritic cells and cytotoxic T cells, delaying pancreatic tumour growth (110). In another example, autologous membrane antigen–laden LNPs (C5) derived from surgically resected colorectal tumours exhibited potent antitumour activity and, when combined with PD-1 blockade, significantly increased remission rates (111).

A liposomal nanovaccine carrying the CD155 gene and modified with Lycium barbarum polysaccharides (LBP-CD155L NVs) improved both prophylactic and therapeutic efficacy in CRC models (112). PLGA-based nanovaccines co-loaded with STING agonists and tumour antigens enhanced lymph node targeting, dendritic cell maturation, and CD8^+^ T-cell activation, suppressing colorectal tumour growth (113). SeaMac nanoparticles—engineered to self-adjuvant antigen presentation—further improved antitumour responses (114). Peptide-based supramolecular nanocarriers enabled tumour-microenvironment-restricted STING agonist release, driving systemic immunity (115).

Gold and silica-based inorganic nanovaccines provide an alternative platform. AuNP-based vaccines promoted photothermal and immunostimulatory activity, leading to CD8^+^ T-cell activation and improved survival in CRC models (116). Silica nanoparticle–based vaccines enhanced T-cell infiltration and tumour control in preclinical colorectal settings (117).

### Cytokine nanodelivery

**Figure 6:**
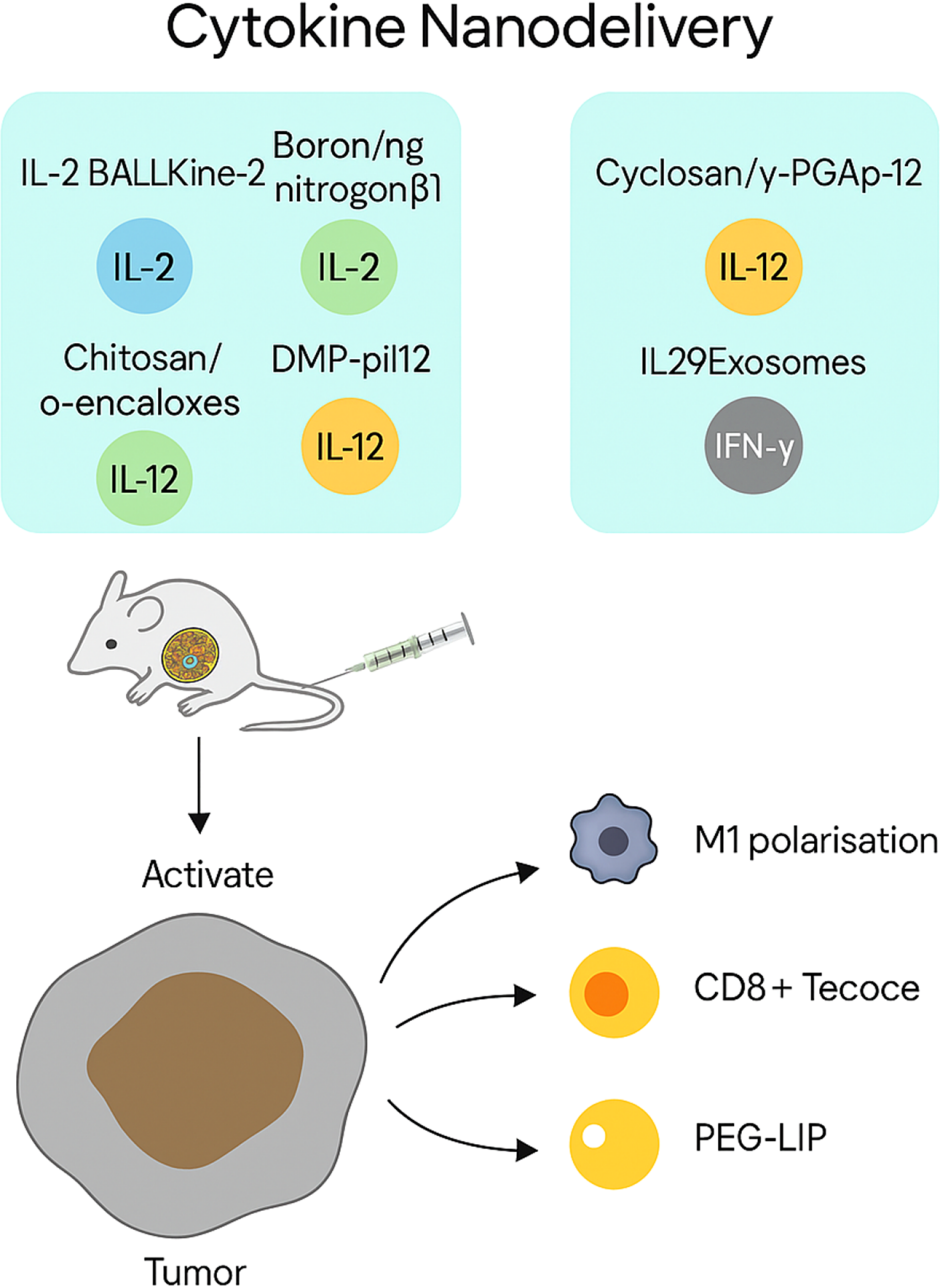
Cytokine nanodelivery for tumour-targeted immune activation. The figure illustrates representative nanocarriers used to deliver cytokines—including IL-2, IL-12, IL-29, and IFN-γ—directly to tumour sites. Platforms such as porous nanoparticles (BALLKine-2), boron–nitrogen nanoparticles, and PEGylated liposomes enable prolonged circulation, controlled release, and reduced systemic toxicity. These systems activate CD8^+^ T cells and dendritic cells, and reprogramme macrophages towards an M1 phenotype, collectively enhancing antitumour immunity.

Nanocarriers permit localised cytokine administration with reduced systemic toxicity (**Figure 6**). IL-2-loaded porous nanoparticles (BALLkine-2) prolonged circulation and reduced toxicity versus free IL-2 (118). Cyclodextrin nanoplexes co-delivering 5-FU and IL-2 demonstrated superior anticancer efficacy in CRC models (119). Another strategy used boron–nitrogen coordinated nanoparticles to improve IL-2 pharmacokinetics and potentiate immune responses in colon cancer, particularly when paired with CDK4/6 inhibitors (120).

IL-12 encapsulation via nanoparticles led to enhanced tumour accumulation and reduced off-target toxicity (121). Extending this concept, a self-stabilising chitosan/poly-(glutamic acid) nanoplatform co-loading doxorubicin and IL-12 achieved prolonged circulation, preferential intratumoural release, and macrophage re-education toward an M1 phenotype, resulting in marked suppression of H22 hepatocellular carcinoma with minimal systemic toxicity (122). The DMP-pIL12 gene delivery system significantly inhibited tumour growth by inducing apoptosis, reducing angiogenesis, and halting proliferation in subcutaneous and peritoneal CRC models (123). A folate-targeted lipoplex (F-PLP/pIL12) delivering IL-12 plasmid DNA resulted in 56.6% tumour inhibition and reduced malignant ascites and tumour burden (124).

IL-29-loaded exosomes showed sustained cytokine release and effective tumour targeting *in vitro* (125). IFN-γ–loaded PEGylated liposomes enhanced macrophage M1 polarisation and accumulated efficiently in colon tumours, suppressing growth in murine models (126).

## Clinical translation of nanomedicine-based therapies in gastrointestinal malignancies

The integration of nanomedicine with immunotherapy has yielded promising antitumour activity in gastrointestinal malignancies, with some agents already entering clinical evaluation. Notably, Abraxane, a nanoparticle albumin-bound formulation of paclitaxel, has been tested in combination with PD-1 blockade across multiple oesophageal and pancreatic cancer trials, consistently demonstrating encouraging response rates and prolonged survival outcomes. Similarly, mRNA nanovaccines—delivered via lipid nanoparticles—have shown robust immunogenicity in early-phase studies in PDAC and metastatic gastrointestinal cancers, offering a platform for personalised immune activation in the adjuvant setting. (**Table 2**)

**Table 2:** Representative clinical trials of nanomedicine-enabled immunotherapy in gastrointestinal cancers.

Nanoformulation	Immunotherapy modality	Trial comparison/control group	Cancer type	Phase	Key outcome	Reference
Abraxane^®^(nab-paclitaxel)	PD-1 inhibitor (camrelizumab)	Camrelizumab + nab-paclitaxel + cisplatin vs. chemo alone	Locally advanced ESCC	III	pCR 28.0% vs. 15.4% (HR for pCR improvement)	(127)
mRNA nanovaccine (autogene cevumeran)	Personalised neoantigen vaccine	Single-arm trial (no control group)	Pancreatic ductal adenocarcinoma	I/II	Induced neoantigen-specific T cells in ~50% of patients	(128)

Several other nanotherapeutics, including Doxil and Onivyde, have demonstrated meaningful cytotoxic efficacy in gastric and pancreatic cancers, respectively. However, these formulations were primarily optimised for chemotherapeutic delivery rather than immunomodulation, and clinical data supporting their synergy with immune checkpoint inhibitors or cellular therapies remain limited. Their inclusion here underscores the therapeutic value of nanomedicine in GI oncology, while highlighting opportunities for further development in immune-based strategies.

### Abraxane

Abraxane, a nanoparticle albumin-bound (nab) formulation of paclitaxel, offers enhanced drug solubility, tumour penetration, and reduced reliance on toxic solvents (129). In advanced oesophageal squamous cell carcinoma (ESCC), combination regimens incorporating Abraxane and PD-1 blockade have demonstrated promising clinical outcomes. Specifically, camrelizumab combined with Abraxane and carboplatin achieved a complete response (CR) rate of 9.8% in patients with advanced ESCC (130), and was associated with 2-year recurrence-free survival (RFS) and overall survival (OS) rates of 67.9% and 78.1%, respectively (131). In another study, intensive induction therapy using camrelizumab with Abraxane and capecitabine resulted in CR and major pathological response (MPR) rates of 33.3% and 64.3%, respectively (132). A phase 2 trial evaluating tislelizumab, a PD-1 inhibitor, in combination with carboplatin and Abraxane, reported MPR and pathological complete response (pCR) rates of 57.5% and 40% in patients with resectable ESCC (133).

In pancreatic cancer, multimodal strategies incorporating Abraxane have also been explored. A combination of CAR T cells targeting EGFR, Abraxane, and cyclophosphamide was deemed safe and induced disease stabilisation and expansion of central memory T cells in patients with metastatic pancreatic cancer, with a median OS of 4.9 months (134). In a phase 1/2 trial, dendritic cell vaccination combined with gemcitabine and Abraxane yielded a median progression-free survival (PFS) of 8.1 months and OS of 15.1 months, without severe adverse events (135). Another phase 1b/2 study assessed a CD40 agonist (sotigalimab) in combination with gemcitabine, Abraxane, and nivolumab, reporting an objective response rate (ORR) of 58% in pancreatic adenocarcinoma (136).

Beyond pancreatic and oesophageal cancers, Abraxane has been investigated in biliary tract cancers (BTCs). When added to the standard gemcitabine–cisplatin regimen, nab-paclitaxel improved outcomes, yielding a median PFS of 11.8 months and OS of 19.2 months, with a disease control rate (DCR) of 84% and ORR of 45% (137). A separate study evaluating nab-paclitaxel plus capecitabine as first-line therapy for advanced or metastatic BTCs reported an ORR of 23.3% and a DCR of 69.8% (138).

However, efficacy in colorectal malignancies appears limited. In a single-arm phase II study, nab-paclitaxel monotherapy demonstrated minimal activity in heavily pretreated metastatic colorectal cancer (mCRC) (139). Similarly, a phase II trial of nab-paclitaxel in CIMP-high mCRC and refractory small bowel adenocarcinoma reported no significant clinical benefit (140).

### mRNA nanovaccines

Lipid nanoparticles (LNPs), composed of a homogeneous lipid core, have emerged as leading non-viral vectors for nucleic acid delivery. Their clinical success in mRNA-based COVID-19 vaccines has accelerated interest in applying this platform to cancer immunotherapy (141, 142). The appeal of mRNA nanovaccines lies in their ability to elicit transient yet potent antigen expression, making them suitable not only for infectious disease prophylaxis but also for applications in cancer vaccination, protein replacement, and gene editing. However, the intrinsic instability of naked mRNA—due to susceptibility to enzymatic degradation and hydrolysis—necessitates protection via encapsulation. LNPs safeguard mRNA from extracellular RNases while facilitating intracellular delivery and endosomal escape (143).

A recent study employed tumour-infiltrating lymphocyte analysis to identify immunogenic mutations in metastatic gastrointestinal cancers. By concatenating validated neoantigens, predicted epitopes, and driver mutations into a single mRNA construct, researchers generated personalised cancer vaccines. The vaccine was well tolerated and induced neoantigen-specific T cell responses, including T cell receptors targeting KRAS^G12D. However, no objective tumour responses were observed in the four patients treated (144). These findings suggest that although mRNA vaccines are immunogenic, combinatorial strategies involving checkpoint inhibition or adoptive T cell transfer may be required to achieve therapeutic benefit.

In a separate phase I study, a personalised uridine mRNA–lipoplex vaccine (autogene cevumeran) was synthesised in real time from resected pancreatic ductal adenocarcinoma (PDAC) tissue. Vaccination expanded neoantigen-specific T cell populations in 50% of treated patients, demonstrating feasibility and immunogenicity in the adjuvant setting (128).

### Doxil

Doxil, a PEGylated liposomal formulation of doxorubicin, was the first nanomedicine approved for oncological use and remains a benchmark in nanoparticle-based chemotherapy (145). Although extensively validated for tumour cytoreduction, it was not originally engineered for immunomodulatory synergy, and its role in immune-based strategies remains to be fully elucidated. By prolonging systemic circulation and enhancing tumour accumulation via the enhanced permeability and retention (EPR) effect, Doxil offers a means to reduce cardiotoxicity while preserving antitumour efficacy.

In a phase II randomised trial comparing two regimens for advanced gastric cancer, a triplet combination of 5-fluorouracil (5-FU), cisplatin, and Doxil (arm A) was evaluated against a standard regimen containing 5-FU, cisplatin, and mitomycin-C (arm B). Arm A achieved a significantly higher overall response rate (ORR) of 64.1% versus 38.5% in arm B (P=0.041). Median time to tumour progression (TTP) was 7.93 vs 5.14 months (P=0.04), and overall survival (OS) was 12.1 vs 8.3 months (P=0.02), favouring the Doxil-containing regimen (146). Another phase II study investigated a four-drug combination including pegylated liposomal doxorubicin, mitomycin C, infusional 5-FU, and sodium folinic acid in patients with advanced gastric cancer. This regimen demonstrated an ORR of 47%, despite the cohort comprising predominantly elderly patients at moderate to high baseline risk (147).

In a phase I trial involving patients with upper gastrointestinal malignancies, preliminary antitumour activity was observed with the same four-drug regimen. Notably, responses were recorded in pretreated pancreatic cancer and untreated gastric cancer, supporting further investigation of Doxil-based combinations in this setting (148).

### Onivyde

Irinotecan, a topoisomerase I inhibitor, is an integral component of the FOLFIRINOX regimen, currently established as first-line therapy for pancreatic ductal adenocarcinoma (PDAC) in multiple international guidelines. However, its efficacy in second-line settings remains limited, and few randomised trials have conclusively defined its value beyond frontline therapy. The liposomal formulation of irinotecan (nal-IRI) exemplifies nanotechnology’s potential to enhance therapeutic efficacy without exacerbating systemic toxicity (149).

In the phase 3 PAN-HEROIC-1 trial, irinotecan hydrochloride liposome (HR070803) combined with 5-fluorouracil and leucovorin significantly improved median overall survival in patients with locally advanced or metastatic PDAC following gemcitabine failure, compared with placebo (150). In a phase 1/2 trial of nal-IRI plus S-1 in gemcitabine-refractory metastatic PDAC, the median overall survival reached 10.3 months, and a confirmed partial response was achieved in 20.4% of patients (151). In Japan, multiple studies have evaluated the safety and efficacy of nal-IRI-based regimens in post-gemcitabine PDAC. One phase 2 trial confirmed the tolerability of nal-IRI with fluorouracil and leucovorin in Japanese patients with unresectable disease (152). Another randomised phase 2 trial comparing nal-IRI plus 5-FU/LV versus 5-FU/LV alone showed improved objective response rate (18% vs 0%) and a median overall survival of 6.3 months in the experimental arm, although OS in the control arm was not reached at the time of analysis (153).

Beyond pancreatic cancer, nal-IRI is being explored in other gastrointestinal malignancies. In RAS wild-type metastatic colorectal cancer, a phase II study (CRACK trial, cohort B) investigated a combination of cetuximab retreatment, camrelizumab, and liposomal irinotecan. This regimen yielded an objective response rate of 25%, a disease control rate of 75%, and median progression-free and overall survival times of 6.9 and 15.1 months, respectively (154). In locally advanced rectal cancer, liposomal irinotecan is being incorporated into multimodal neoadjuvant strategies. A recent phase 2 trial examined nal-IRI in combination with 5-FU, leucovorin, and oxaliplatin, followed by chemoradiotherapy in a watch-and-wait programme. Among patients achieving clinical complete response (cCR), the median disease-free survival (DFS) was not reached after 32 months of follow-up, with 1-, 2-, and 3-year DFS rates of 90.0%, 80.0%, and 80.0%, respectively (155).

## Future perspectives and conclusion

Nanoformulations hold great promise for onco-immunotherapy, owing to their biocompatibility, stability, and capacity for precise tumour targeting (156). Yet, their translation into the clinic faces several key hurdles. First, nanoparticles are inherently more complex than small molecules: parameters such as size, composition, surface chemistry, and shape all influence *in vivo* behaviour (157), and these properties can shift upon protein corona formation, complicating PK–PD relationships. Second, the rapid clearance of nanoparticles by the reticuloendothelial system (RES) limits tumour bioavailability and risks off-target toxicity in the liver and spleen; although PEGylation and size minimisation can mitigate this (158), complete RES evasion remains elusive. Third, heterogeneous tumour architectures and variable EPR effects mean that neither active nor passive targeting guarantees uniform delivery (159); responsive nanocarriers that release payloads under specific TME cues (e.g., pH, enzyme activity) offer a way forward but require precise tuning of size and charge to optimise penetration and retention (160). Finally, nanotoxicology—encompassing genotoxicity, immunotoxicity, and organ‐specific adverse effects—remains under-characterised; systematic, standardised assays in physiologically relevant models are essential before first-in-human studies (161).

In addition, the practical feasibility and commercial viability of nanomedicines depend upon reproducible synthesis methods, cost-effective manufacturing, stringent quality control, and scalable production capabilities (162, 163). The inherent complexity of nanoformulations often renders large-scale production challenging, necessitating simplified yet robust synthetic approaches that maintain consistency between batches (164). Rigorous examination of toxicity profiles, biosafety risks, and nanocarrier–tissue interactions is paramount, as discrepancies between *in vitro* and *in vivo* outcomes frequently occur during preclinical development (165–167). Furthermore, robustly designed clinical trials remain critical, demanding comprehensive evaluation not only of efficacy but also mechanisms of action, toxicity, survival outcomes, and clinically relevant adverse events (168).

Beyond these considerations, nano-immunotherapeutics must also navigate stringent regulatory and good-manufacturing-practice (GMP) complexities. Regulatory guidelines from the US FDA (Drug Products, Including Biological Products, that Contain Nanomaterials) and EMA (Reflection Paper on nanotechnology-based medicinal products) emphasise early identification and stringent control of critical quality attributes (CQAs), including particle size, polydispersity, surface charge, encapsulation efficiency, residual solvents, endotoxin limits, sterility, and *in vitro* release characteristics, aligned with quality-by-design frameworks (ICH Q8–Q10) (169). Continuous-flow or microfluidic manufacturing processes, while enabling precise control of particle characteristics, introduce GMP-specific challenges related to cleaning validation, in-line monitoring, and batch consistency (170, 171). Real-world cases of approved nanomedicines underscore these complexities: PEGylated liposomal doxorubicin (Doxil^®^) faced significant manufacturing deviations necessitating revalidation and bioequivalence studies (172, 173); patisiran (Onpattro^®^), the first siRNA LNP therapeutic, leveraged GMP-compliant microfluidic platforms for consistent production (170); and the rapid deployment of mRNA–LNP COVID-19 vaccines highlighted additional challenges in RNA stability, cold-chain logistics, and impurity control (174, 175). Consequently, systematic toxicological assessment and rigorous biosafety evaluations remain prerequisites for the successful translation of nanomedicine into clinical practice.

Emerging clinical and preclinical data support the synchronised use of nano-formulated immunotherapeutics with checkpoint inhibitors, adoptive cell therapies, or TLR/STING agonists to overcome tumour immunosuppression. Neoadjuvant trials in colorectal cancer—such as AVANA, combining chemoradiation with pembrolizumab—have achieved pathological complete response rates up to 23% and major pathological response rates of 61.5% (176), highlighting the feasibility of early‐stage intervention and on-treatment biomarker assessment. Incorporating nanomedicines into these settings may further amplify immune activation, improve margin clearance, and allow rapid pharmacodynamic readouts to refine dosing and sequence.

While intravenous lipid nanoparticles have dominated the field, alternative administration routes better aligned with tumour biology warrant vigorous exploration. Oral or intraluminal delivery of nanoparticles—designed to withstand gastrointestinal barriers—can directly target colorectal lesions, as reviewed by Pavitra et al. in orally administrable formulations achieving enhanced mucosal uptake and reduced systemic toxicity (177). Similarly, intraperitoneal delivery may increase regional exposure in peritoneal metastases of gastric or ovarian origin. Development of selective‐organ‐targeting LNPs, which exploit lipid composition to redirect payloads to specific organs, further exemplifies how chemical tuning can achieve precision biodistribution.

The complexity of nanoparticle disposition—shaped by protein corona formation, RES clearance, and tumour penetration—demands embedded PK–PD and immune readouts in early clinical trials. High‐resolution imaging (e.g., PET‐labeled nanoparticles), single‐cell RNA sequencing of serial biopsies, and circulating immune profiling will enable correlations between formulation parameters and therapeutic response. Machine‐learning models trained on these multimodal datasets can then predict optimal nanoparticle designs and dosing regimens, closing the loop between experimental insight and clinical application (178).

In concert, these strategies—rational combination regimens, tailored delivery approaches, and rigorous translational science—will be essential to overcome current barriers and deliver precision‐engineered nanotherapies with durable efficacy and safety in gastrointestinal malignancies.
